# Prevalence of toxigenic fungi and mycotoxins in Arabic coffee (*Coffea arabica*): Protective role of traditional coffee roasting, brewing and bacterial volatiles

**DOI:** 10.1371/journal.pone.0259302

**Published:** 2021-10-29

**Authors:** Wadha Al Attiya, Zahoor Ul Hassan, Roda Al-Thani, Samir Jaoua

**Affiliations:** Environmental Science Program, Department of Biological and Environmental Sciences, College of Arts and Science, Qatar University, Doha, Qatar; Universita degli Studi di Pisa, ITALY

## Abstract

Fungal infection and synthesis of mycotoxins in coffee leads to significant economic losses. This study aimed to investigate the prevalence of toxigenic fungi, their metabolites, and the effect of traditional roasting and brewing on ochratoxin A (OTA) and aflatoxins (AFs) contents of naturally contaminated coffee samples. In addition, *in vivo* biocontrol assays were performed to explore the antagonistic activities of *Bacillus simplex* 350–3 (BS350-3) on the growth and mycotoxins synthesis of *Aspergillus ochraceus* and *A*. *flavus*. The relative density of *A*. *niger*, *A*. *flavus*, *Penicillium verrucosum* and *A*. *carbonarius* on green coffee bean was 60.82%, 7.21%, 3.09% and 1.03%, respectively. OTA contents were lowest in green coffee beans (2.15 μg/kg), followed by roasted (2.76 μg/kg) and soluble coffee (8.95 μg/kg). Likewise, AFs levels were highest in soluble coffee (90.58 μg/kg) followed by roasted (33.61 μg/kg) and green coffee (9.07 μg/kg). Roasting naturally contaminated coffee beans at three traditional methods; low, medium and high, followed by brewing resulted in reduction of 58.74% (3.50 μg/kg), 60.88% (3.72 μg/kg) and 64.70% (4.11 μg/kg) in OTA and 40.18% (34.65 μg/kg), 47.86% (41.17 μg/kg) and 62.38% (53.73 μg/kg) AFs contents, respectively. Significant inhibitions of AFs and OTA synthesis by *A*. *flavus* and *A*. *carbonarius*, respectively, on infected coffee beans were observed in presence of *Bacillus simplex* BS350-3 volatiles. Gas chromatography mass spectrochemistry (GC-MS/MS) analysis of head-space BS350-3 volatiles showed quinoline, benzenemethanamine and 1-Octadecene as bioactive antifungal molecules. These findings suggest that marketed coffee samples are generally contaminated with OTA and AFs, with a significant level of roasted and soluble coffee contaminated above EU permissible limits for OTA. Further, along with coffee roasting and brewing; microbial volatiles can be optimized to minimize the dietary exposure to mycotoxins.

## Introduction

Coffee, due to its physiological effects and organoleptic characteristic is widely consumed as non-alcoholic beverage with high economic impact. Like other crops, coffee beans are also prone to microbial infections during production, processing and storage stages [1, 2]. Prevalence of toxigenic *Aspergillus* and *Penicillium* spp., on coffee beans has been reported in coffee growing as well as non-growing parts of the world [3, 4]. These fungal infections at favorable environmental conditions generally leads to the accumulation of mycotoxins [5–7]. Ochratoxin A (OTA) and aflatoxin B1 (AFB1), which are known for their nephrotoxic and hepatoxic activities, respectively, are most commonly found on the coffee beans [8]. European Union (EU) has set maximum permissible limit for OTA [9] in roasted (5 μg/kg) and soluble (10 μg/kg) coffee, while no such regulations exists for green coffee beans [10]. Unlike OTA, there are no set regulations by EU for aflatoxins (AFs) levels in the green, roasted and soluble coffee.

Apart from coffee production and proper post-harvest management (sorting, grading, storage temperature and humidity); preparation of coffee plays an important role in controlling the levels of mycotoxins in final drink. In particular, a significant reduction in OTA content occur during coffee roasting [11–14], grinding [15] and brewing [16]. Such coffee preparation techniques are highly variable and sometime strictly traditional to develop specific color and aroma, that affect differentially the level of mycotoxins [14, 16]. It is, however, reasonable to assume higher mycotoxins in ready-to-drink coffee prepared according to traditional Arabic method compared to dark roast coffee.

There are several approaches to minimize the fungal infection on food crops and agricultural products. Among these are the implementation of improved preharvest managemental strategies such as using resistant crop varieties, crop rotation, proper sowing and harvesting timings, etc. Other practices include the use of chemical pesticides and fungicides to control the fungal infections [17]. Taking into account the arising human health issues due to residual transfer of fungicides in food chain and emergence of fungicide resistance in fungal strains, research focusing on developing novel strategies ensuring safety to the environment and nontarget species is increasing [18, 19]. In this connection, biological control approaches with the application of environment-friendly yeast and bacterial strains and their molecules are getting increasing popularity [20–22]. The possible mechanism of action of these biologics include competing for the nutrients and space, emission of antifungal diffusible and volatile molecules, provoking resistance in crops against fungal attack, etc. [23, 24].

In this study, focus was made to investigate; a) the prevalence of toxigenic fungi and mycotoxins in green, roasted and soluble coffee marketed in Qatar; b) the individual and combined effects of traditional coffee roasting (low, medium and high) and brewing on the reduction of OTA and AFs in naturally contaminated coffee beans, and c) the biological control activities of a novel bacterium *Bacillus simplex* (QBS350-3) on the growth and mycotoxins synthesis by *A*. *carbonarius* and *A*. *flavus* on infected coffee beans.

## Material and methods

### Isolation and identification of mycotoxigenic fungal communities from marketed coffee

In total, 25 coffee samples {green beans (n = 08), roasted beans (n = 08) and soluble powder (n = 09)} were procured from the markets in Qatar. All the samples were transported to lab in sterile air-tight bags and stored at 4°C before analysis for fungal contamination and mycotoxins contents. Coffee beans (green and roasted) were disinfected by dipping (1 min) in 5% sodium hypochlorite (NaClO), washed with sterile distilled water and placed on Dichloran-Rose Bengal Chloramphenicol agar (DRBC). Five beans from each sample were placed aseptically on Petri dishes in triplicate. Soluble coffee samples were 1:10 diluted in sterile water, 100 μL of the suspension from each sample was plated on DRBC agar plates in triplicate, and incubated for 7 days at 28°C [25]. The fungal contamination frequency (Fr %) of each coffee sample was calculated as below.


$$Fungal\;contamination\;frequency\;(Fr\;\%)=\frac{No.of\;infected\;beans}{Total\;No.of\;beans}X\;100$$

Isolated fungal colonies were purified by obtaining single spore culture [26]. For the morphological identification of *Aspergillus* and *Penicillium* fungi, the isolates were transferred to identification media; malt extract agar (MEA), Czapek yeast extract agar (CYA), and dichloran-glycerol agar (DG18) following the scheme described by Pitt and Hocking [25]. The recipes for the preparation of these media are described by Hassan et al. [27]. The relative density of identified fungal species were calculated using the following equation.


$$Relative\;density\;(RD\;\%)\;of\;fungal\;specie=\frac{No.of\;isolates\;of\;a\;species}{Total\;No.of\;fungal\;isolates}x\;100$$

### Analysis of mycotoxins in the coffee samples

Ochratoxin A (OTA) and aflatoxins (AFs) extraction from all the coffee samples was performed according to the recommendation of ELISA kits manufacturer (R-Biopharm, Germany). Briefly, all the coffee beans samples were ground to powder using blender. To 5 g of sample, 12.5 mL of 70% methanol was added, mixed and filtered using Whatman No. 1 filter papers. Filtrate, after dilution were applied to ELISA wells (RIDASCREEN^®^ OTA ELISA kits or RIDASCREEN^®^ Aflatoxins total ELISA kits). TECAN Sunrise^TM^ ELISA plate reader (Männedrof, Switzerland) was used to measure the absorbance at 450 nm and data was analyzed using RIDASOFT^®^ Win (Z9996, R-Biopharm, Germany).

### Effect of traditional coffee roasting and brewing on mycotoxin contents

In order to explore the individual and combined effect of traditional coffee roasting and brewing, contaminated coffee beans were roasted, ground and brewed according to traditional Arabic methods. For this purpose, green coffee beans samples naturally contaminated at three levels each for OTA (10.38, 3.10 and 2.91 μg/kg) and AFs (87.40, 83.31 and 87.78 μg/kg) were chosen. The beans were roasted using three traditional Qatari coffee roasting methods, locally designated as 1.0 (low roast), 1.5 (medium roast) and 2.0 (high roast). Low roast scheme involved initial heating the beans at 140°C (10 min), then 180°C (2 min), followed by slow cooling in wooden trays. In case of medium roast, after initial heating at 140°C (10 min), beans were roasted at 180°C (5 min), and then at 200°C (2 min), before cooling down at room temperature. High roast scheme (R2.0) started with heating the bean at 140°C (10 min), followed by roasting at 180°C (5 min), and finally at 210°C (5 min), before cooling. During all the roasting steps, beans were kept rotating with electric spinner. All the roasted samples along with their unroasted (control) were ground to power using coffee blender. A portion of ground samples was analyzed for the OTA and AFs contents as described above. The remaining portions were subjected to traditional brewing before analysis for mycotoxins levels. The effect of traditional roasting (different schemes) and brewing on the reduction of mycotoxins levels was calculated by the following formulas:

$$Reduction\;of\;mycotoxin\;(\%)\;due\;to\;coffee\;roasting\;(a)=\frac{\left(x-y\right)}{x}\;x\;100$$

$$Total\;reduction\;of\;mycotoxin\;(\%)\;after\;roasting\;and\;brewing\;(b)=\frac{\left(x-z\right)}{x}\;x\;100$$

Reductionofmycotoxin(%)duetocoffeebrewing(c)=b−a

whereas:

*x* = Initial level of mycotoxin in coffee samples
*y* = Level of mycotoxin after coffee roasting
*z* = Level of mycotoxin after brewing the roasted samples

### Biocontrol activities of *Bacillus simplex* (BS350-3) on fungal infected coffee beans

In order to investigate the *in vivo* antifungal activity *B*. *simplex* (BS350-3), co-incubation experiments were performed in Petri dishes (90 x 15 mm) divided in three compartments. Prior to start the experiment, one compartment of Petri dish was filled with 8 mL of tryptic soy agar (TSA, Sigma-Aldrich, USA) and was spread inoculated with 35 μL of *B*. *simplex* (BS350-3) cell suspension. Plates were incubated for 24 hrs. In the control Petri dishes, TSA filled compartment was maintained without any bacterial inoculation. In the other two compartments, surface disinfected (dipped in 5% NaOCl solution for 1 min and then washed) dried green coffee beans were placed. Beans in one compartment were point inoculated with 10 μL of spore suspension (1 x 10^4^/mL) from 7-day old culture of *A*. *carbonarius* or *A*. *flavus*, while in the other compartment beans were kept as uninoculated control. After placing the covers, Petri dishes were sealed with 2 layers of Parafilm^®^ along with one cover of sealing tape to allow the exposure of fungal spores to BS350-3 volatiles. In control plates, fungal spores on coffee beans were exposed to volatiles emitted from TSA alone. At day 11 (of incubation at 28°C), Petri dishes were opened, and coffee beans were collected, grind to powder and analyzed for the levels of OTA and/or AFs. This experiment was repeated thrice with a minimum of three replicates each time.

### GC-MS based analysis of bacterial volatiles organic compounds (VOCs)

Bioactive volatile molecules emitted by antagonistic *B*. *simples* 350–3 were identified through Gas chromatography mass spectrometry (GC-MS). For this purpose, method described by Saleh et al. [23] for capturing the bacterial volatiles on activated charcoal and eluting in dichloromethane was applied. The mass-spectra of unknown compounds were submitted to Wiley and NIST libraries for identification of molecules [22]. Control media flasks were maintained by incubating TSB without bacteria.

### Statistical analysis

The data on fungal contamination of coffee beans was calculated by the equations given above and presented as isolation frequency and relative density. Mycotoxin contents of the coffee samples were calculated in μg/kg and presented as mean ± SD as well as in ranges (min.–max.). Statistical package SPSS was used to record the effect of traditional roasting, brewing and bacterial volatiles on mycotoxins contents. Mycotoxin reduction in percent as well as actual values were calculated. Analysis of variance (ANOVA) test was used and followed by Duncan’s multiple range test (DMRT) for *post-hoc* multi-comparison.

## Results and discussion

### Mycobiota of marketed coffee samples

**Fig 1:**
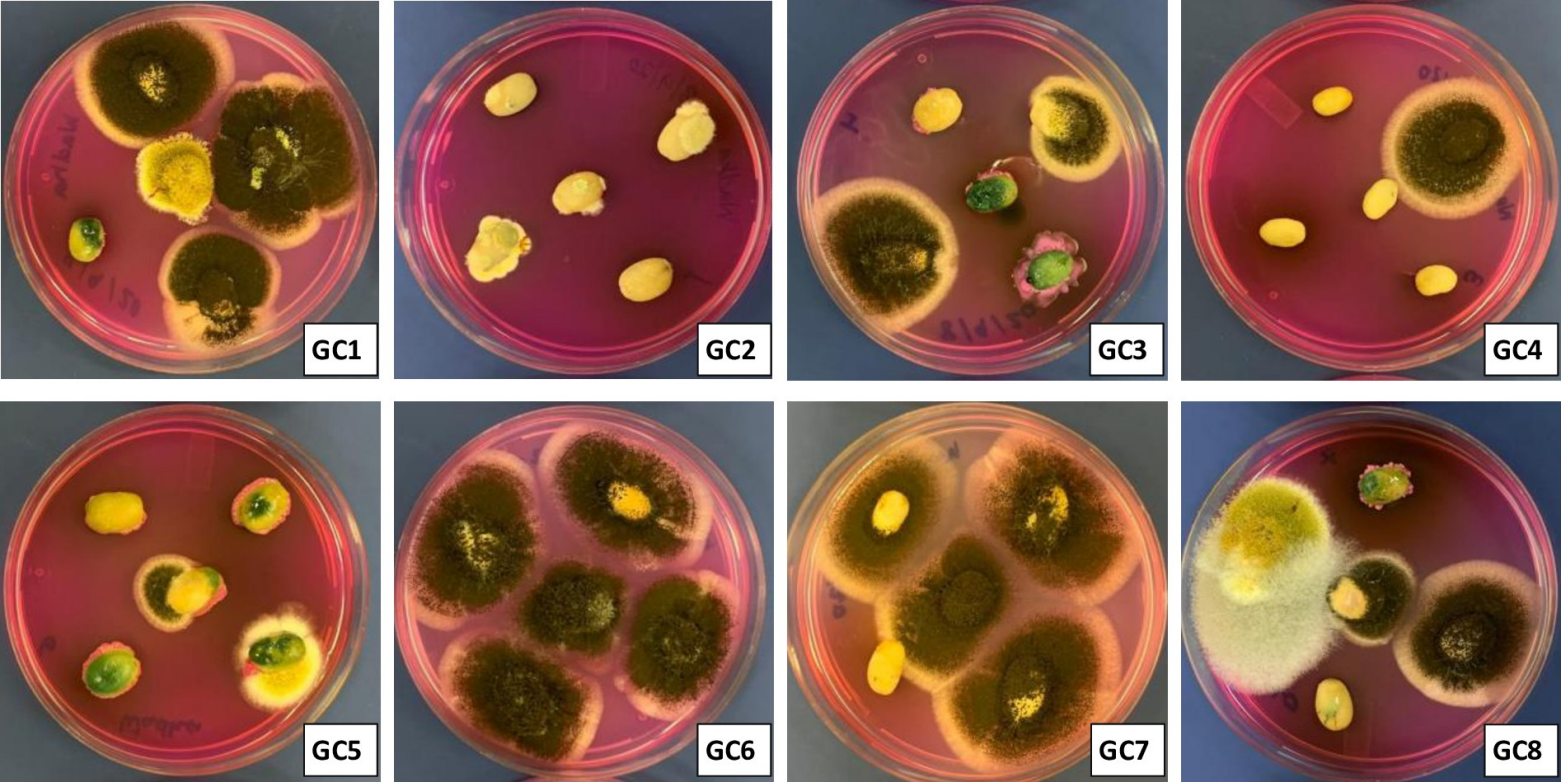
Isolation of fungi on DRBC agar from green coffee (GC) beans samples. Isolation of fungi on Dichloran Rose Bengal Chloramphenicol (DRBC) agar from green coffee (GC) beans samples. All green coffee beans samples yielded fungal communities, while none of roasted and soluble coffee samples showed fungal infection.

Incidence of filamentous fungi is an important problem of coffee production and may occur in pre-harvest field conditions and/or post-harvest storage and handling [2, 28]. In the present study, no fungal colonies were isolated from roasted and soluble coffee samples. This might be associated with the killing of fungal spores during coffee roasting and further sterilized air-tight packaging of soluble coffee samples which prevented the fungal contamination. However, unlike our findings, Casas-Junco et al. [3] in Mexico reported ochratoxigenic fungi as well as OTA in the marketed roasted coffee samples. In general, fungal spore and mycelium are sensitive to coffee roasting temperature, while a significant portion of already synthesized mycotoxins are retained unchanged. The presence of viable fungal spore in the study of Casas-Junco et al. [3] suggests a possible fungal contamination during post-roasting processing and storage stages, and the availability of favorable environmental conditions promoted mycotoxin synthesis. On the other hand, in the present study none of the green coffee samples were free from fungal contamination (Fig 1). The fungal isolation frequency (Fr %) on green coffee beans samples, calculated on the basis of infected beans vs total tested beans for each sample ranged from 73% - 100% (Fig 1). The relative density of *A*. *niger*, *A*. *flavus*, *A*. *tamarii*, *Penicillium verrucosum*, *A*. *carbonarius* and *Fusarium* spp., were 60.1, 7.2, 5.2, 3.1, 1.0 and 10.3, respectively. The remaining 12.4% represents the unidentified fungal species. In line with the present study, in Brazil, green coffee beans from conventional and organic cultivation system were equally contaminated with ochratoxigenic fungi, including *A*. *niger* and *A*. *ochraceus* [29]. Although not detected is the present study, the ochratoxigenic *A*. *westerdijkiae* may also contaminate green coffee samples, leading to synthesis of significant amount of OTA [30].

### Comparative mycotoxins contents in green, roasted and ready-to-use coffee samples

All the tested coffee samples in this study were contaminated by OTA; lowest levels were detected in green coffee beans (2.15 ± 0.51 μg/kg), followed by roasted coffee (2.76 ± 1.46 μg/kg) and soluble coffee (8.95 ± 3.80 μg/kg). Such higher levels of OTA in soluble coffee samples, as compared to roasted coffee beans have previously been reported in Chile [12]. There are no established regulations for OTA permissible levels in green coffee beans in most of the EU countries [10]. However, some countries such as Italy, Finland and Greece have legal OTA limits of 8, 10 and 20 μg/kg, respectively, for green coffee beans [16]. Considering these statutory limits of European Union (EU) members countries, in the present study, all the green coffee bean samples showed OTA levels within the safer limits (Table 1). In Brazil, Taniwaki et al. [30] reported higher levels (11.3–25.7 μg/kg) of OTA compared to our findings. Likewise, Nakajima et al. [31] in Japan found 30% of the tested green coffee samples contaminated with OTA at 0.1–17.4 μg/kg. In Switzerland, in a recent study on green coffee [32], OTA was found in 45% of the samples at maximum levels of 12.2 μg/kg. These levels were much higher compared to 1.75–3.34 μg/ kg in the present study (Table 1), probably due to the occurrence of OTA producing species such as *A*. *westerdijkiae* [30]. In roasted coffee beans, EU set a maximum permissible limit of OTA as 5 μg/kg [9]. In the present study, 2 samples of roasted coffee (25%) showed the OTA levels above the permissible levels.

**Table 1:** Mycotoxins levels (μg/kg) in marketed coffee samples.

Sample type	Parameter	Mycotoxins (μg/kg)	
		Aflatoxins	Ochratoxin A
Green coffee beans (n = 8)	Mean ± SD	9.07 ± 5.26	2.15 ± 0.51
	Range	0–14.50	1.75–3.34
Roasted coffee beans (n = 8)	Mean ± SD	33.61 ± 18.83	2.76 ± 1.46
	Range	7.55–67.42	1.68–6.14
Soluble coffee (n = 9)	Mean ± SD	90.58 ± 39.08	8.95 ± 3.80
	Range	29.01–136.08	2.52–12.74

Nielsen et al. [33] found OTA in about half (n = 57) of the tested roasted coffee samples, with 5 samples above the EU permissible limit. The maximum OTA level of 21 μg/kg they found in the roasted coffee was much higher if compared to the present study (6.14 μg/kg). However, soluble coffee showed highest OTA contamination levels with 44% of the tested samples displaying OTA contents above the EU permissible limits of 10 μg/kg. Unlike our findings, Nielsen et al, [33] in Brazil, found OTA in all the 25 soluble coffee samples within the EU permissible limits. In Italy [34], all 96% of the OTA positive soluble (0.32–6.40 μg/kg) coffee samples were within EU permissible limits.

In case of aflatoxins (AFs), highest contamination was found in soluble coffee samples (90.58 ± 39.08 μg/kg) followed by roasted (33.61 ± 18.83 μg/kg) and green coffee (9.07 ± 5.26 μg/kg). Levels of AFs contaminations in coffee is not monitored throughout the world including EU. In the early research it was believed that the presence of caffeine in the coffee prevents the accumulation of AFs by the *Aspergillus* spp. Later on, it has been shown that AFs can be found in coffee samples from the market as well as storage facilities [32, 35]. Soliman [35] found AFs in green and roasted coffee at 4.28 μg/kg and 2.85 μg/kg, respectively. Compared to roasted coffee (54.5%), a higher number of green coffee samples (76.5%) were contaminated with AFs. In contrast, in the present study, 87.5% of green coffee beans and 100% of each roasted as well as soluble coffee sample were contaminated with AFs. Likewise, in the present study levels of AFs were higher in roasted beans compared to the green coffee beans, which is opposite to the findings of Soliman [35]. In Switzerland [32], AFs were detected in green coffee at much lower concentration of 1.2 μg/kg. These differences might be linked to the factors including storage conditions, duration, toxigenic activities of infecting fungal strain, nature and grade of coffee beans, etc.

### Effect of traditional coffee roasting and brewing on mycotoxins levels

#### Effect on OTA contents

**Fig 2:**
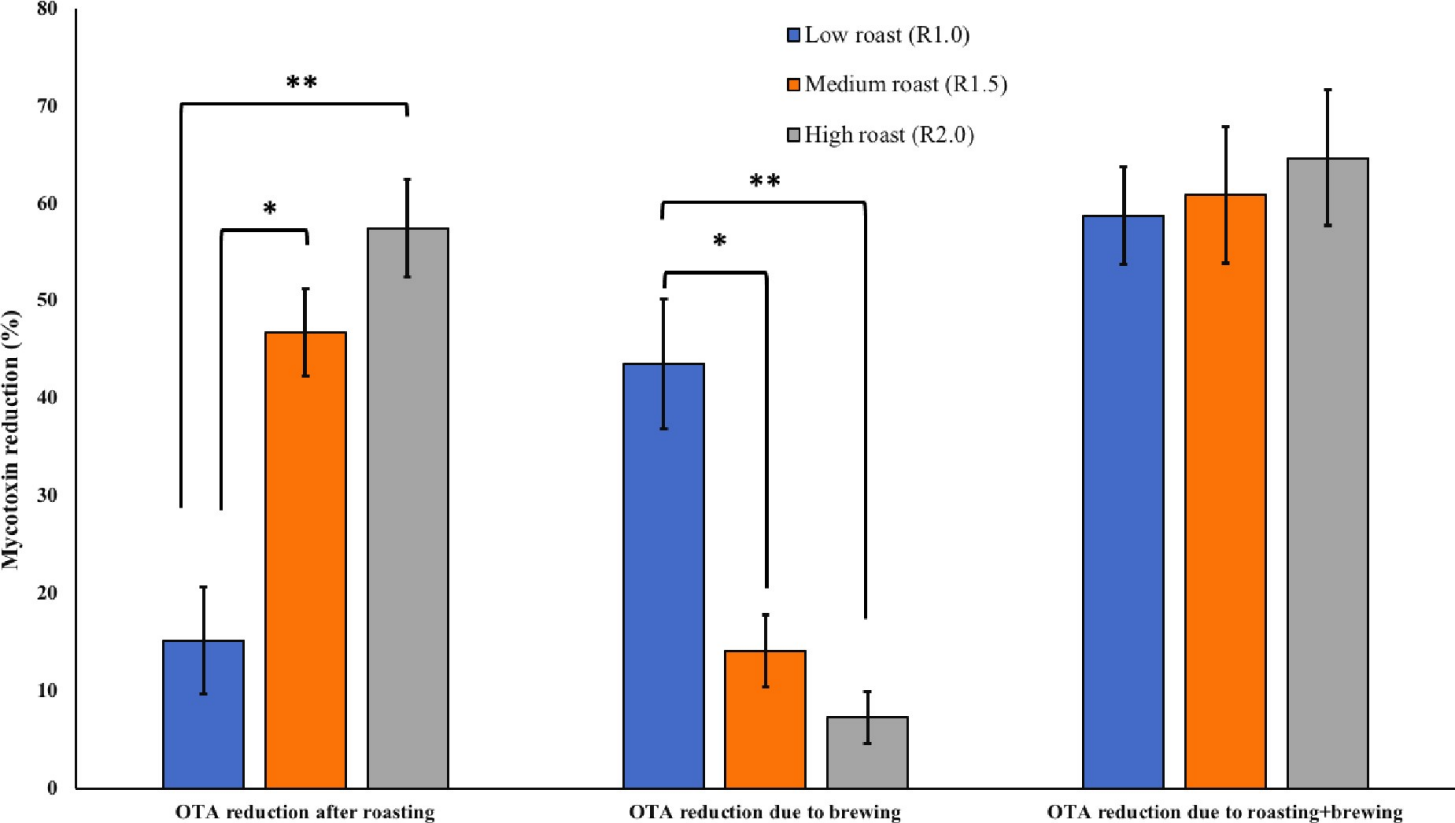
Effect of traditional roasting and brewing on OTA reduction (%) in naturally contaminated coffee beans. Effect of traditional roasting and brewing on OTA reduction (%) in naturally contaminated coffee beans. Roasting OTA contaminated coffee beans at traditionally low, medium and high methods resulted in 15.17% (0.61 μg/kg), 46.78% (2.99 μg/kg) and 57.43% (3.82 μg/kg) reduction in mycotoxin contents, respectively. Brewing of OTA coffee samples already roasted at low, medium and high schemes resulted in further 43.57%, 14.11% and 7.28% reduction in mycotoxin, respectively.

Roasting OTA contaminated coffee beans at traditionally low, medium and high methods resulted in 15.17% (0.61 μg/kg), 46.78% (2.99 μg/kg) and 57.43% (3.82 μg/kg) reduction in mycotoxin contents, respectively (Fig 2).

Medium and high roasting showed significantly higher degradation of OTA as compared to low roasting method. In line with our findings, Ferraz et al. [11] recorded 53% decline in the OTA contents of the coffee samples treated at 180°C for 12 min. Oliveira et al. [15], reported on average 64.51%, 88.90% and 96.38% reduction in OTA levels in the coffee samples roasted at light (205°C for 13.5 min), medium (217°C for 14 min) and dark (224°C for 24 min) levels, respectively. The higher OTA reduction % achieved by Oliveira et al. [15] compared to our study is due their higher roasting temperature (224°C) and duration of up to 14 min, whereas in our case coffee samples were roasted maximum at 210°C. Roasting conditions for the coffee are strictly traditional and according to the consumer interests. In most of the Arabian countries, coffee is roasted at comparatively lower temperature to maintain lighter color of the final drink. Such roasting at lower temperature, e.g., 180°C (10 min) resulted in a lower reduction (31%) of OTA contents [13]. Brewing of OTA contaminated coffee samples roasted at low, medium and high schemes resulted in 43.57% (2.89 μg/kg), 14.11% (0.73 μg/kg) and 7.28% (0.29 μg/kg) reduction in mycotoxin contents. Brewing of low roasted coffee showed significantly higher reduction in OTA compared to brewing of medium and high roasted samples. Considering a significant effect of brewing on mycotoxin contents, in the final drink, the levels of toxins observed in the soluble and roasted sample will fall within the maximum permissible limits. Pérez et al. [14] evaluated three brewing methods on OTA reduction and founds espresso coffee maker as most effective with 49.8% reduction in mycotoxins levels. In the present study, the ready to drink (after brewing) samples showed 58.74% (3.50 μg/kg), 60.88% (3.72 μg/kg) and 64.70% (4.11 μg/kg) reduction in the initial OTA contents of coffee samples roasted at three methods, low, medium and high schemes, respectively.

#### Effect on AFs levels

**Fig 3:**
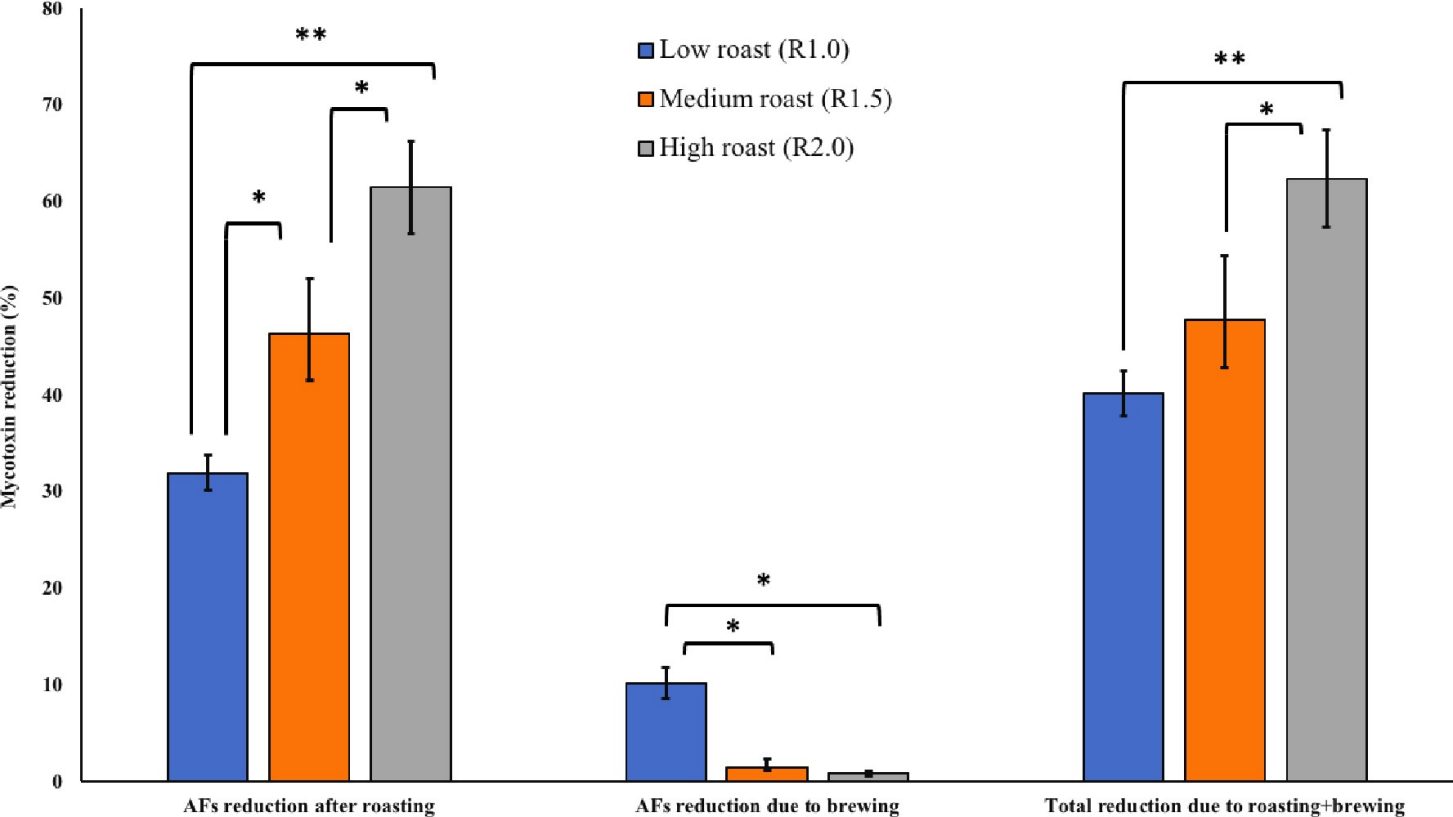
Effect of traditional roasting and brewing on AFs reduction (%) on naturally contaminated coffee beans. Effect of traditional roasting and brewing on AFs reduction (%) on naturally contaminated coffee beans. The combined effect of roasting and brewing was 40.18% (34.65 μg/kg), 47.86% (41.17 μg/kg) and 62.38% (53.73 μg/kg) reduction in the AFs levels in the coffee samples roasted at traditionally low, medium and high methods, respectively.

The roasting conditions such as temperature and duration play significant role in reducing the mycotoxins contents in coffee beans [35–37]. In the present study, roasting coffee beans at three traditional methods significantly reduced AFs contents in the naturally contaminated samples (Fig 3). Low (R1.0), medium (R1.5) and high (R2.0) roast resulted in 31.98% (27.52 μg/kg), 46.36% (39.89 μg/kg) and 61.52% (52.98 μg/kg) reduction in AFs levels in the contaminated beans, respectively. There is scarce information on the effect of coffee roasting and brewing on aflatoxins contents. Soliman [35] observed 55.9% reduction in aflatoxins contents of coffee samples traditionally roasted at 180°C. In our study, the effect of medium and high roasting was significantly higher in AFs reduction as compared to low roasting. Likewise, high roast (R2.0) resulted in significant (*p* < 0.01) reduction in AFs levels compared to medium roast (R1.5). Unlike the present study, Humaid et al. [36] observed a lesser reduction (20%) in AFs contents of the Yemeni coffee samples. Roasting coffee at 200°C [37], resulted in 93% (light roast) and 100% (dark roast) reduction in AFB1 contents. Brewing of already roasted coffee samples at low, medium and high methods resulted in further 10.19% (7.13 μg/kg), 1.50% (1.28 μg/kg) and 0.86% (0.74 μg/kg) reduction in AFs levels. Effect of brewing in reduction of AFs levels was significantly higher (*p* < 0.05) on coffee samples roasted at low method compared to those roasted at medium and high schemes.

The combined effect of roasting and brewing was 40.18% (34.65 μg/kg), 47.86% (41.17 μg/kg) and 62.38% (53.73 μg/kg) reduction in the AFs levels in the coffee samples roasted at traditionally low, medium and high methods, respectively. Coffee beans roasted (alone) at high temperature showed a significant reduction in AFs contents compared to cumulative effect of roasting (medium and low) and brewing. In all cases, the effect of roasting (at any of three methods) on reducing the AFs contamination was significantly higher compared to brewing alone.

### *In vivo* biocontrol of mycotoxins on coffee beans

**Fig 4:**
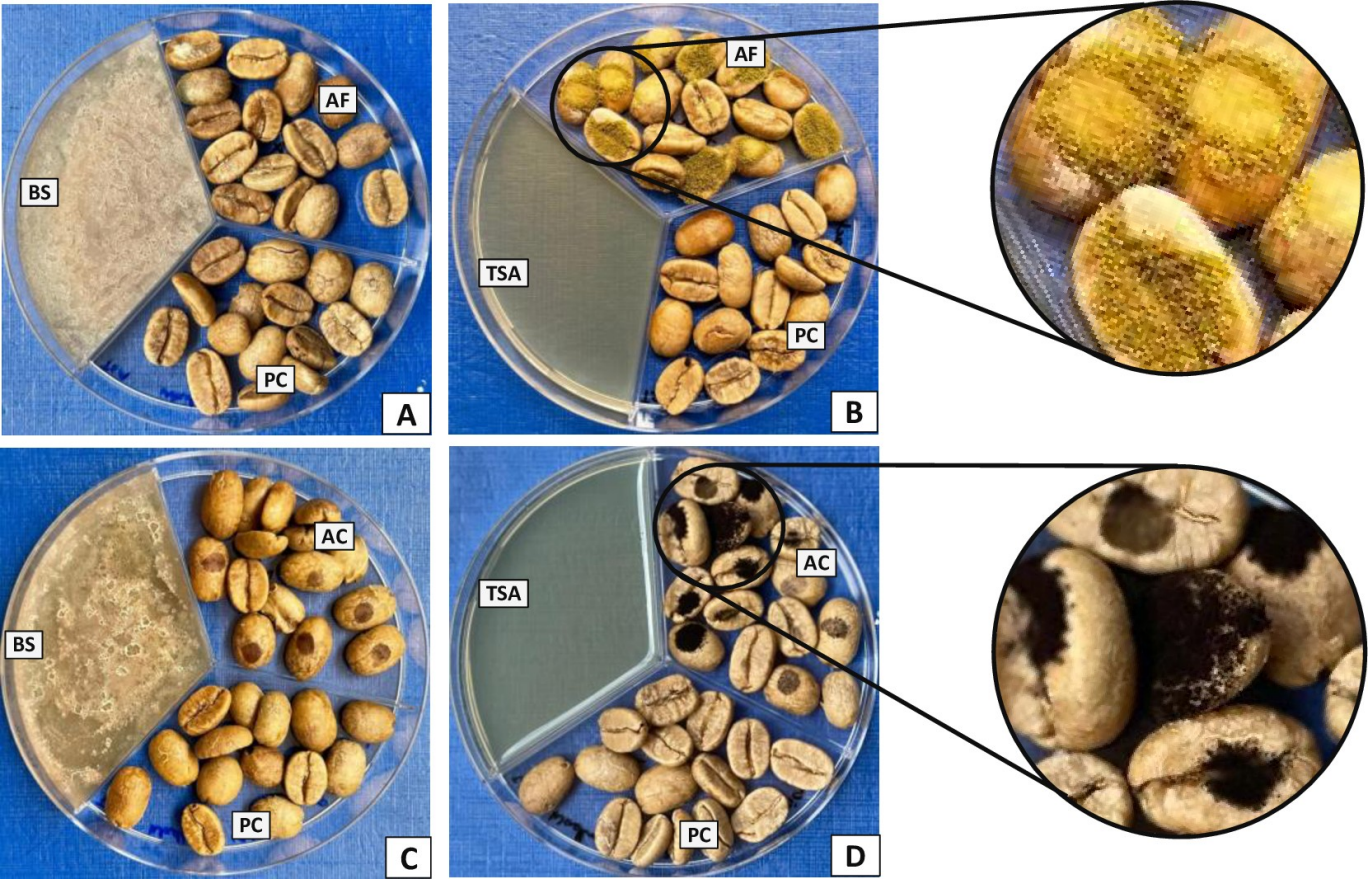
*In vivo* antagonistic activity of *Bacillus simplex* 350–3 volatiles against the growth of toxigenic fungi on infected coffee beans. There was a significant growth of *A*. *flavus* (B) and *A*. *carbonarius* (D) on coffee beans in the absence of *B*. *simplex* volatile, compared to complete inhibition of fungi in A and B in the presence of bacterial volatile. BS (*Bacillus simplex* on tryptic soy agar); AF (coffee beans infected with *Aspergillus flavus*); PC (Plain coffee beans); AC (coffee beans infected with *Aspergillus carbonarius*); TSA (tryptic soy agar without any bacteria).

Biological control of mycotoxins in agriculture and food industry is getting popularity because of its safety and lesser chances of emergence of resistance in target fungal strains. In previous studies it has been reported that volatiles emitted from yeasts [21, 29, 38], bacteria [22, 23] and fungi [39] possess substantial activities to inhibit the spread and mycotoxin production by toxigenic fungi. In the present *in vivo* study, toxigenic fungi on coffee beans were exposed to *B*. *simplex* 350–3 volatiles to evaluate the effect on fungal growth and mycotoxin synthesis. In the presence of BS350-3 VOC’s, the growth of *A*. *flavus* (Fig 4A) and *A*. *carbonarius* (Fig 4C) were completely inhibited, while in the absence of *B*. *simplex* there were significant growth of *A*. *flavus* (Fig 4B) and *A*. *carbonarius* (Fig 4D) on the infected coffee beans. The observed inhibition of fungal growth on infected coffee beans may be associated with inhibitory role of bacterial molecules on the fungal spores germination [40] or suppression of gene responsible for vegetative fungal growth [38]. A significant inhibition of toxigenic fungi and mycotoxin synthesis on coffee beans have been achieved with the application antagonistic yeasts [40, 41], and fungal strains [39].

Mycotoxins contents of the infected coffee beans incubated in the *B*. *simplex* volatile’s environment showed a significant inhibitory role of bacterial volatiles (Table 2). AFs contents in the coffee beans infected with *A*. *flavus* and incubated in the absence of BS350-3 volatiles were significantly higher (63.60 ± 8.52 μg/kg) compared to beans incubated in the presence of bacterial volatile (26.65 ± 3.33 μg/kg). Likewise, *A*. *carbonarius* was able to accumulate significantly higher OTA (4.13 ± 0.81 μg/kg) in the environment free from the BS350-3 volatiles, compared to the levels noted in the presence of bacterial volatiles (1.88 ± 0.33 μg/kg). These finding suggest significant inhibitory role of BS350-3 volatile on the spread and toxin production by the fungi. In line with these findings, exposure to *B*. *megaterium* (BM344-1) volatiles [23] resulted in complete suppression in AFs and OTA production by *A*. *flavus* and *A*. *carbonarius*, respectively. Likewise, on maize ears BM344-1 volatiles showed significant reduction in AFs accumulation by toxigenic *A*. *flavus*. In our previous study [22], *B*. *licheniformis* 350–2 volatiles mixture comprising mainly on 3-methyl-1-butanol significantly suppressed AFs and OTA synthesis by *A*. *flavus* and *A*. *carbonarius*, respectively. It is, however, unclear whether the observed inhibition of mycotoxins synthesis, in the present study is directly associated with the pronounced inhibition of fungal growth or suppression of cluster genes involved in mycotoxins biosynthetic pathways [39].

**Table 2:** Effect of bacterial volatiles on the inhibition of mycotoxins synthesis by toxigenic fungi on coffee beans.

Treatment	Aflatoxin (μg/kg)	OTA (μg/kg)
Control (plane coffee beans)	30.05 ± 3.27^b^	1.39 ± 0.12^b^
*Fungal infected coffee beans	63.60 ± 8.52^a^	4.13 ± 0.81^a^
Fungal infected coffee incubated in the presence of *B*. *simplex*	26.65 ± 3.33^b^	1.88 ± 0.33^b^

* Coffee beans were infected with *A*. *flavus* to evaluate aflatoxin production and with *A*. *carbonarius* to evaluate OTA production

### Analysis of bioactive bacterial volatile molecules

GC-MS/MS analysis of *B*. *simplex* 350–3 emitted volatiles showed the presence of quinoline, benzenemethanamine and 1-Octadecene (Table 3). The lack of these molecules in the control flasks (TSB media alone) showed that antifungal activity of *B*. *simplex* was either alone or combined effect of these 3 compounds. The antifungal activities of compounds identified in BS350-3 and their derivatives have been reported by many researchers [42, 43]. Naureen et al. [42] isolated *Lysinibacillus sphaericus* ZA9 from maize rhizosphere producing quinoline alkaloid which possess significant activity against plant pathogenic fungal species including *Aspergillus* species. Vinale et al. [43] isolated 4-(hydroxymethyl)-quinoline in fungal filtrate showing significant inhibition of pathogenic *Sclerotinia sclerotiorum* and *Pythium irregulare*, and weaker activity against *Rhizoctonia solani*. Similarly, Talie et al. [44] reported significant antifungal activity of a mushroom extract containing 1-Octadecene along with other compounds against *Penicillium*, *Aspergillus* and *Alternaria* species, mainly by inhibition of fungal spore germination. These findings suggest potential application of a novel *B*. *simplex* 350–3 in preservation and storage of food and agriculture products.

**Table 3:** Analysis of *B*. *simplex* 350–3 volatiles through GC-MS/MS.

S. No.	Molecule	Retention time (min)	Peak area (%)
1	Quinoline	18.42	2.44
2	Benzenemethanamine	24.98	5.58
3	1-Octadecene	34.38	4.48

## Conclusion

In this study, we have reported the prevalence of toxigenic fungi, OTA and AFs in marketed coffee samples in Qatar. The presence of AFs at high levels in the roasted and soluble coffee, highlight the need to set maximum permissible limit for this mycotoxin on an analogy of OTA. There is a significant effect of traditional coffee roasting and brewing on reducing the OTA and AFs contents. These methods can be optimized to reduce the dietary exposure of mycotoxins through coffee and their impact on human health. There was significant reduction in the spread of *A*. *ochraceus* and *A*. *flavus* and mycotoxins synthesis on coffee when incubated in the environment of *B*. *simplex* 350–3 volatiles. Application of *B*. *simplex* 350–3 in coffee production can replace the use of synthetic fungicides and their transmission to food chain.

## Supporting information

S1 Data (XLSX)

## References

[1] Bucheli P, Taniwaki MH. Research on the origin, and on the impact of post-harvest handling and manufacturing on the presence of ochratoxin A in coffee. Food Additives and Contaminants. 2002;19: 655–665. doi: 10.1080/02652030110113816 12113660

[2] Silva CF, Batista LR, Schwan RF. Incidence and distribution of filamentous fungi during fermentation, drying and storage of coffee (Coffea arabica L.) beans. Braz J Microbiol. 2008;39: 521–526. doi: 10.1590/S1517-838220080003000022 24031259 PMC3768428

[3] Casas-Junco PP, Ragazzo-Sánchez JA, Ascencio-Valle F de J, Calderón-Santoyo M. Determination of potentially mycotoxigenic fungi in coffee (Coffea arabica L.) from Nayarit. Food Sci Biotechnol. 2018;27: 891–898. doi: 10.1007/s10068-017-0288-7 30263816 PMC6049681

[4] Viegas C, Pacífico C, Faria T, de Oliveira AC, Caetano LA, Carolino E, et al. Fungal contamination in green coffee beans samples: A public health concern. Journal of Toxicology and Environmental Health, Part A. 2017;80: 719–728. doi: 10.1080/15287394.2017.1286927 28548622

[5] Bokhari FM. Mycotoxins and Toxigenic Fungi in Arabic Coffee Beans in Saudi Arabia. 2007; 12.

[6] Bokhari F, Aly MM. Trials towards reduction of fungal growth and aflatoxin G1 production in Arabic coffee using different additives. Afr. J. Food Sci. 3 2009; 68–76.

[7] Rezende E de F, Borges JG, Cirillo MÂ, Prado G, Paiva LC, Batista LR. Ochratoxigenic fungi associated with green coffee beans (Coffea arabica L.) in conventional and organic cultivation in Brazil. Braz J Microbiol. 2013;44: 377–384. doi: 10.1590/S1517-83822013000200006 24294225 PMC3833131

[8] Leitão AL. Occurrence of Ochratoxin A in Coffee: Threads and Solutions—A Mini-Review. Beverages. 2019;5: 36. doi: 10.3390/beverages5020036

[9] Commission Regulation (EC) No 1881/2006 of 19 December Setting maximum levels for certain contaminants in foodstuffs. *Of- ficial J Eur Communities* 20/12/2006. L364/5.

[10] Commission Regulation (EC) No 105/2010 of 5 February Amend- ing Regulation (EC) n° 105/2010 as regards Ochratoxin A toxins. *Official J Eur Communities* 6/02/2010. L35/7.

[11] Ferraz MBM, Farah A, Iamanaka BT, Perrone D, Copetti MV, Marques VX, et al. Kinetics of ochratoxin A destruction during coffee roasting. Food Control. 2010;21: 872–877. doi: 10.1016/j.foodcont.2009.12.001

[12] Galarce-Bustos O, Alvarado M, Vega M, Aranda M. Occurrence of ochratoxin A in roasted and instant coffees in Chilean market. Food Control. 2014;46: 102–107. doi: 10.1016/j.foodcont.2014.05.014

[13] Nehad EA, Farag MM, Kawther MS, Abdel-Samed AKM, Naguib K. Stability of ochratoxin A (OTA) during processing and decaffeination in commercial roasted coffee beans. Food Additives and Contaminants. 2005;22: 761–767. doi: 10.1080/02652030500136852 16147432

[14] Pérez De Obanos A, González-Peñas E, López De Cerain A. Influence of roasting and brew preparation on the ochratoxin A content in coffee infusion. Food Additives and Contaminants. 2005;22: 463–471. doi: 10.1080/02652030500090042 16019819

[15] Oliveira G, da Silva DM, Alvarenga Pereira RGF, Paiva LC, Prado G, Batista LR. Effect of different roasting levels and particle sizes on ochratoxin A concentration in coffee beans. Food Control. 2013;34: 651–656. doi: 10.1016/j.foodcont.2013.06.014

[16] Vieira T, Cunha S, Casal S. Mycotoxins in Coffee. Coffee in Health and Disease Prevention. Elsevier; 2015. pp. 225–233. doi: 10.1016/B978-0-12-409517-5.00025–5

[17] Chandler D. The consequences of the ‘cut off’ criteria for pesticides: Alternative methods of cultivation. Directorate General Internal Policies of the Union (2008). Retrieved from http://www.europarl.europa.eu/RegData/etudes/note/join/2008/408962/IPOL-AGRI_NT(2008)408962_EN.pdf

[18] Köhl J, Kolnaar R, Ravensberg WJ. Mode of Action of Microbial Biological Control Agents Against Plant Diseases: Relevance Beyond Efficacy. Front Plant Sci. 2019;10: 845. doi: 10.3389/fpls.2019.00845 31379891 PMC6658832

[19] Tsitsigiannis I, Antoniou P, Tjamos C. Biological control strategies of mycotoxigenic fungi and associated mycotoxins in Mediterranean basin crops. Phytopathologia Mediterranea. 2012;51: 17.

[20] Gonçalves A, Gkrillas A, Dorne JL, Dall’Asta C, Palumbo R, Lima N, et al. Pre- and Postharvest Strategies to Minimize Mycotoxin Contamination in the Rice Food Chain: Mycotoxins in the rice food chain…. Comprehensive Reviews in Food Science and Food Safety. 2019;18: 441–454. doi: 10.1111/1541-4337.12420 33336939

[21] Tilocca B, Balmas V, Hassan ZU, Jaoua S, Migheli Q. A proteomic investigation of Aspergillus carbonarius exposed to yeast volatilome or to its major component 2-phenylethanol reveals major shifts in fungal metabolism. International Journal of Food Microbiology. 2019;306: 108265. doi: 10.1016/j.ijfoodmicro.2019.108265 31325815

[22] Ul Hassan Z, Al Thani R, Alnaimi H, Migheli Q, Jaoua S. Investigation and Application of *Bacillus licheniformis* Volatile Compounds for the Biological Control of Toxigenic *Aspergillus* and *Penicillium* spp. ACS Omega. 2019;4: 17186–17193. doi: 10.1021/acsomega.9b01638 31656892 PMC6811857

[23] Saleh AE, Ul-Hassan Z, Zeidan R, Al-Shamary N, Al-Yafei T, Alnaimi H, et al. Biocontrol Activity of *Bacillus megaterium* BM344-1 against Toxigenic Fungi. ACS Omega. 2021;6: 10984–10990. doi: 10.1021/acsomega.1c00816 34056251 PMC8153935

[24] Tilocca B, Cao A, Migheli Q. Scent of a Killer: Microbial Volatilome and Its Role in the Biological Control of Plant Pathogens. Front Microbiol. 2020;11: 41. doi: 10.3389/fmicb.2020.00041 32117096 PMC7018762

[25] Pitt JI, Hocking A.D. Fungi and food spoilage. (3rd ed.) 2009. New York: Springer

[26] Balmas V, Migheli Q, Scherm B, Garau P, O’Donnell K, Ceccherelli G, et al. Multilocus phylogenetics show high levels of endemic fusaria inhabiting Sardinian soils (Tyrrhenian Islands). Mycologia. 2010;102: 803–812. doi: 10.3852/09-201 20648748

[27] Hassan ZU, Al-Thani RF, Migheli Q, Jaoua S. Detection of toxigenic mycobiota and mycotoxins in cereal feed market. Food Control. 2018;84: 389–394. doi: 10.1016/j.foodcont.2017.08.032

[28] Adhikari M, Isaac EL, Paterson RRM, Maslin MA. A Review of Potential Impacts of Climate Change on Coffee Cultivation and Mycotoxigenic Fungi. Microorganisms. 2020;8: 1625. doi: 10.3390/microorganisms8101625 33096901 PMC7590209

[29] Zeidan R, Ul-Hassan Z, Al-Thani R, Balmas V, Jaoua S. Application of Low-Fermenting Yeast Lachancea thermotolerans for the Control of Toxigenic Fungi Aspergillus parasiticus, Penicillium verrucosum and Fusarium graminearum and Their Mycotoxins. Toxins. 2018;10: 242. doi: 10.3390/toxins10060242 29904020 PMC6024770

[30] Taniwaki MH, Teixeira AA, Teixeira ARR, Copetti MV, Iamanaka BT. Ochratoxigenic fungi and ochratoxin A in defective coffee beans. Food Research International. 2014;61: 161–166. doi: 10.1016/j.foodres.2013.12.032

[31] Nakajima M, Tsubouchi H, Miyabe M, Ueno Y. Survey of aflatoxin B _1_ and ochratoxin A in commercial green coffee beans by high‐performance liquid chromatography linked with immunoaffinity chromatography. Food and Agricultural Immunology. 1997;9: 77–83. doi: 10.1080/09540109709354938

[32] Bessaire T, Perrin I, Tarres A, Bebius A, Reding F, Theurillat V. Mycotoxins in green coffee: Occurrence and risk assessment. Food Control. 2019;96: 59–67. doi: 10.1016/j.foodcont.2018.08.033

[33] Nielsen KF, Ngemela AF, Jensen LB, de Medeiros LS, Rasmussen PH. UHPLC-MS/MS Determination of Ochratoxin A and Fumonisins in Coffee Using QuEChERS Extraction Combined with Mixed-Mode SPE Purification. J Agric Food Chem. 2015;63: 1029–1034. doi: 10.1021/jf504254q 25553918

[34] Vecchio A, Mineo V, Planeta D. Ochratoxin A in instant coffee in Italy. Food Control. 2012;28: 220–223. doi: 10.1016/j.foodcont.2012.04.029

[35] Soliman KM. Incidence, Level, and Behavior of Aflatoxins during Coffee Bean Roasting and Decaffeination. J Agric Food Chem. 2002;50: 7477–7481. doi: 10.1021/jf011338v 12452679

[36] Humaid AAH, Alghalibi SMS, Ahmed E. Aflatoxins and Ochratoxin A Content of Stored Yemeni Coffee Beans and Effect of Roasting on Mycotoxin Contamination. Molecular Microbiology. 2019;2:11–21.

[37] Micco C, Miraglia M, Brera C, Desiderio C, Masci V. The effect of roasting on the fate of aflatoxin B1 in artificially contaminated green coffee beans. Mycotoxin Research. 1992;8: 93–97. doi: 10.1007/BF03192222 23606005

[38] de Almeida ÂB, Corrêa IP, Furuie JL, de Farias Pires T, do Rocio Dalzoto P, Pimentel IC. Inhibition of growth and ochratoxin A production in Aspergillus species by fungi isolated from coffee beans. Braz J Microbiol. 2019;50: 1091–1098. doi: 10.1007/s42770-019-00152-9 31515726 PMC6863313

[39] Farbo MG, Urgeghe PP, Fiori S, Marcello A, Oggiano S, Balmas V, et al. Effect of yeast volatile organic compounds on ochratoxin A-producing Aspergillus carbonarius and A. ochraceus. International Journal of Food Microbiology. 2018;284: 1–10. doi: 10.1016/j.ijfoodmicro.2018.06.023 29990634

[40] Masoud W, Kaltoft CH. The effects of yeasts involved in the fermentation of Coffea arabica in East Africa on growth and ochratoxin A (OTA) production by Aspergillus ochraceus. International Journal of Food Microbiology. 2006;106: 229–234. doi: 10.1016/j.ijfoodmicro.2005.06.015 16213049

[41] Souza ML, Passamani FRF, Ávila CL da S, Batista LR, Schwan RF, Silva CF. Use of wild yeasts as a biocontrol agent against toxigenic fungi and OTA production. Acta Sci Agron. 2017;39: 349. doi: 10.4025/actasciagron.v39i3.32659

[42] Naureen Z, Rehman NU, Hussain H, Hussain J, Gilani SA, Al Housni SK, et al. Exploring the Potentials of Lysinibacillus sphaericus ZA9 for Plant Growth Promotion and Biocontrol Activities against Phytopathogenic Fungi. Front Microbiol. 2017;8: 1477. doi: 10.3389/fmicb.2017.01477 28861045 PMC5563071

[43] Vinale F, Ghisalberti EL, Flematti G, Marra R, Lorito M, Sivasithamparam K. Secondary metabolites produced by a root-inhabiting sterile fungus antagonistic towards pathogenic fungi. Letters in Applied Microbiology. 2010;50: 380–385. doi: 10.1111/j.1472-765X.2010.02803.x 20156309

[44] Talie MD, Wani AH, Lone BA, Bhat MY. Chemical composition and antifungal activity of essential oil of rhizopogon species against fungal rot of apple, J. App. Biol. Sci. 2020;14: 296–308.

